# Efficient Support Vector Regression for Wideband DOA Estimation Using a Genetic Algorithm

**DOI:** 10.3390/s25092915

**Published:** 2025-05-05

**Authors:** Yonghong Zhao, Gang Zheng, Junlong Wang, Jisong Liu, Shuxin Dong, Jing Xin

**Affiliations:** 1 School of Automation and Information Engineering, Xi’an University of Technology, Xi’an 710048, China; zhaoyh2018@xaut.edu.cn (Y.Z.); 2230321285@stu.xaut.edu.cn (J.W.); 2230320112@stu.xaut.edu.cn (J.L.); 2220321243@stu.xaut.edu.cn (S.D.); xinj@xaut.edu.cn (J.X.); 2 Shaanxi Key Laboratory of Complex System Control and Intelligent Information Processing, Xi’an University of Technology, Xi’an 710048, China

**Keywords:** DOA estimation, broadband signals, machine learning, support vector regression, genetic algorithm

## Abstract

High-precision direction of arrival (DOA) of wideband signals is a very important technology in the field of radar and communication. In this work, we propose an efficient support vector regression (SVR) architecture via a genetic algorithm (GA) for wideband DOA estimation, which exhibits high estimation performance and generalization performance. By adopting the two-sided correlation transformation (TCT) algorithm, the network is trained only from reference frequency data to increase the training efficiency. In order to reduce the redundant information in the array covariance matrix and lower the dimensionality of the input features, the array covariance matrix at a single frequency point is preprocessed according to its conjugate symmetry and elemental characteristics, and the dimensionality-reduced input features are obtained. Specifically, the dimensionality of the input features does not increase with the number of sub-bands when dealing with broadband signals or ultra-broadband signals, which can significantly reduce the training time of the model and the storage capacity of the system. The increased performance of the proposed algorithm is highly desirable in resource-constrained scenarios, and the experimental results demonstrate the efficiency and superiority of the proposed network compared with existing methods.

## 1. Introduction

Direction of arrival (DOA) estimation is a crucial part of array signal processing that is widely used in radar, sonar, navigation, communication, and various other applications [1,2,3]. The main goals of DOA estimation are to improve the estimation precision and reduce computational complexity simultaneously. After a long period of development, researchers have proposed many DOA estimation methods, such as conventional beamforming (CBF) [4,5,6], multiple signal classification (MUSIC) [7,8,9,10], maximum likelihood estimation (ML) [11,12,13,14], and estimation of signal parameters via rotational invariance techniques (ESPRIT) [15,16,17]. The performance of these methods significantly degrades with a low signal-to-noise ratio (SNR) or a small number of snapshots. Later, many researchers applied sparse representation (SR) [18,19,20] to DOA estimation and achieved good estimation accuracy. However, DOA estimations based on SR rely on prior information about the signals. Additionally, all of the above approaches belong to the category of model-driven approaches and require the establishment of a parametric model between the angle information of incident signals and array outputs. In practical applications, it is difficult to establish a complete and accurate parametric model due to the presence of element errors and mutual coupling effects. Meanwhile, the challenges in achieving high-precision calibration make traditional DOA estimation poorly adaptable to complex electromagnetic environments and reduce the estimation performance.

With the expansion of the field of DOA estimation, more requirements have been imposed for its effectiveness and real-time performance, compelling researchers to continuously seek new technological approaches. Machine learning is a data-driven approach used to learn signal features from massive amounts of data and can transcend model constraints to overcome the limitations of traditional approaches [21]. Now, machine learning theory has been widely applied in DOA estimation. It can be adapted to complex and changing environments through real-time learning and adaptive adjustment [22], thus improving response speed and accuracy to track signal and environmental changes. Furthermore, by integrating modern hardware and optimization strategies during online testing, machine learning approaches can fully harness parallel computing advantages, thereby improving computational efficiency and satisfying real-time performance demands following offline training. The latest research achievements in DOA estimation have provided new ideas for increasing accuracy, robustness, and processing efficiency. One option is to incorporate the You Only Look Once version 3 (YOLOv3) [23] neural network structure into DOA estimation. By framing the problem as a target detection task, this method avoids the typical spectral peak search step, allowing for end-to-end estimation. Another significant research direction is the construction of an iterative neural network based on deconvolved beamforming [24]. This technique reduces the training burden and the need for large-scale datasets. In addition, it has a strong ability to suppress sidelobes and false peaks, especially under low-SNR conditions. As a result, the robustness and accuracy of DOA estimation have greatly improved. Particle swarm optimization (PSO) is employed to fine-tune the weights and thresholds of a backpropagation (BP) neural network [25], thereby enhancing the accuracy and real-time performance of the DOA estimation and improving its adaptability to dynamic environments. To enhance the accuracy of DOA estimation under low-SNR conditions, Guo et al. [26] proposed a DOA estimation method based on support vector regression (SVR) optimized by a genetic algorithm (GA). Leveraging the GA to optimize the SVR parameters during training significantly enhances the model’s generalization capability. To solve the difficulty of artificially adjusting parameters, a single-snapshot DOA estimation approach based on the alternating-direction multiplier method (ADMM) was presented [27]. This algorithm reduced the need for manual intervention by automatically modifying parameters during training, improving the overall efficiency of the estimation process. An unsupervised learning network was developed based on a thresholded Capon spectral weighting penalty [28]. This method is especially useful in high-interference settings, where it increases the robustness and accuracy of the DOA estimation by implementing an adaptive spectral weighting penalty. Zhao et al. [29] introduced a novel deep convolutional neural network named TADCN for 2D-DOA estimation using an L-shaped array. The network incorporates a triple attention mechanism (TAM) to capture channel, height, and width relationships of signal features, improving feature extraction and spatial spectrum quality. However, the aforementioned methods are designed for far-field narrowband signal processing. Wideband signals exhibit different envelopes at each array element, which makes them difficult to directly apply to the DOA estimation of broadband signals. With the advancement of modern communication and radar technologies, narrowband signals increasingly fail to meet practical application requirements, such as those of electronic warfare, spectrum sensing, and through-wall radar imaging. These practical application scenarios have consequently elevated wideband DOA estimation to a critical research area.

The general processing approaches for wideband DOA estimation include the incoherent signal subspace method (ISM) [30,31,32] and the coherent signal subspace method (CSM) [33,34,35]. The ISM algorithm estimates the DOAs of broadband sources by dividing the entire frequency domain into multiple sub-bands, performing narrowband estimation in each frequency band individually, and weighting the spatial spectra in each band. This type of algorithm performs poorly under low-SNR conditions. The CSM algorithm focuses the array covariance matrices of the broadband signals in each frequency band into a specific frequency band and applies narrowband techniques to estimate the spatial spectra in this frequency band. This method offers higher estimation accuracy and a lower resolution threshold and is capable of handling coherent sources. However, its sensitivity to prediction angle deviations means that inaccurate predictions can substantially degrade DOA estimation performance.

In recent years, significant advancements have been made in wideband DOA estimation techniques, driven by their expanding applications across multidisciplinary domains. It is worth mentioning that some model-based methods have achieved good results in the DOA estimation of broadband signals. Zhang et al. conducted extensive research on this topic. A unified DOA estimation model for hybrid analog–digital architectures was proposed [36], accompanied by the derivation of a dynamic maximum likelihood estimator and its Cramér–Rao bound. Recent developments in tensor-based processing have also shown promising results in addressing high-dimensional signal processing challenges. For example, a tensor-decomposition-based channel estimation framework was introduced for extremely large-scale MIMO-OFDM systems with dynamic metasurface antennas [37]. While these model-driven approaches provide a strong theoretical foundation, their reliance on signal assumptions and their computational complexity in dynamic environments significantly constrain their applicability in real-time, adaptive scenarios. In contrast, deep learning methods offer enhanced estimation accuracy and robustness, particularly in complex electromagnetic environments. Among these methods, the integration of the attention mechanism considerably enhances DOA estimation performance [38]. However, the high computing cost of this method limits its application in real-time systems. Convolutional neural networks (CNNs) [39] are widely used for DOA estimation of broadband signals due to their powerful nonlinear mapping capabilities, which enable excellent accuracy. In addition, deep-learning-based approaches [40,41] offer superior robustness and adaptability in wideband DOA estimation, ensuring reliable performance across diverse and challenging environments. These methods are extremely demanding in terms of training data and may be limited in their ability to generalize to unseen scenarios. To address the complexity of wideband signals, a method based on coherent wideband SVR (CWSVR) [42] has been proposed. This method performs well in processing wideband signals. However, as the number of sub-bands grows, the input dimension of the network increases significantly, resulting in a substantial rise in training time. This limits the applicability of the method to large-scale datasets. To address this issue, a novel multiband joint direction-finding SVR model is proposed. The model employs a processed and optimized array covariance matrix as input features, while its parameters are fine-tuned using a genetic algorithm. Then, an effective framework is constructed and applied to DOA estimation. This approach not only improves estimation performance through joint estimation across frequency bands but also effectively reduces model training time. The method proposed in this paper is mainly aimed at the DOA estimation of wideband signals, but it can also be applied to ultra-wideband signals. It is worth noting that broadband signals are defined as signals with a relative bandwidth greater than 10% of the carrier frequency. Ultra-wideband (UWB) signals have a relative bandwidth greater than 20% of the carrier frequency. The contributions of our work are summarized as follows:

- An efficient SVR architecture for wideband DOA estimation via a GA is proposed. It exhibits superior estimation and resolution capabilities, along with strong generalization performance. The results of experiments confirm the superior performance and efficiency of the proposed network compared to typical existing algorithms such as RSS-MUSIC, L1-SVD, and CWSVR.
- We have employed a coherent focusing operation to initially extract the features from the covariance matrix and ensure the preservation of essential signal features. Multiband data are projected onto a designated reference frequency point, which substantially lowers the dimensionality of the input signals to the network. In particular, the dimensionality of the input features does not scale with the number of sub-bands, making the method well suited for broadband and ultra-broadband signals. This not only substantially reduces the model training time and system storage requirements but also makes the method especially beneficial in resource-limited environments.
- The performance of SVR models is highly sensitive to parameter selection, particularly the regularization and kernel function parameters. Here, we employ a GA to optimize the model parameters due to its notable advantages, including global optimization capability, strong adaptability, and effectiveness in mitigating overfitting. The optimized SVR model exhibits improved fitting and generalization performance on the training data, leading to a significant improvement in DOA estimation.

The remainder of this paper is organized as follows. In Section 2, we introduce the mathematical model and theoretical foundations for wideband array signal processing. In Section 3, we present our methodology, including the data preprocessing, parameter optimization, and model construction. In Section 4, the effectiveness of the algorithm is verified through simulation and compared with existing methods. Finally, conclusions are drawn in Section 5.

## 2. Wideband Array Signal Model

Consider a uniform linear array (ULA) with M sensors and _*K*_ wideband signals $s\left(t\right)={\left[s_{1}\left(t\right),\cdots ,s_{K}\left(t\right)\right]}^{T}$ in the far-field from direction $\theta ={\left[\theta_{1},\cdots ,\theta_{K}\right]}^{T}$. Then, the received signal at the _*m*_-th sensor of the ULA can be expressed as

(1) $$x_{m}\left(t\right)=\sum_{k=1}^{K}s_{k}\left(t-\tau_{m,k}\right)+n_{m}\left(t\right),m=1,2,\cdots ,M$$

where ${\left(\cdot \right)}^{T}$ is the transpose operation, $\tau_{m,k}=\left(m-1\right)d\sin\theta_{k}/c$ denotes the time delay of the k-th target at the _*m*_-th sensor relative to the reference sensor, d is the spacing between sensors, _*c*_ is the speed of electromagnetic wave propagation, and $n_{m}\left(t\right)$ denotes the noise observed at the _*m*_-th sensor.

For the whole array, variations in the envelope and phase of the wideband signal across different sensors cannot be ignored. Therefore, it is typically necessary to divide the array output for frequency division processing to establish a frequency-domain model. We transform the temporally sampled data of the M sensors into the frequency domain using a discrete Fourier transform (DFT), which divides it into J sub-bands. Then, the mathematical model of the j-th frequency point is [43]:

(2) $$x\left(f_{j}\right)=A\left(f_{j},\theta \right)s\left(f_{j}\right)+n\left(f_{j}\right),\;j=1,2,\cdots ,J$$

where $x(f_{j})={\left[x_{1}(f_{j}),\cdots ,x_{M}(f_{j})\right]}^{T}$ and $n(f_{j})={\left[n_{1}(f_{j}),\cdots ,n_{M}(f_{j})\right]}^{T}$ denote the Fourier coefficient vector of the array’s received data and noise at the frequency point $f_{j}$, respectively; $x_{m}(f_{j})$ is the Fourier coefficient of the signal $x_{m}\left(t\right)$ received at the _*m*_-th sensor. $s(f_{j})={\left[s_{1}(f_{j}),\cdots ,s_{K}(f_{j})\right]}^{T}$ is the K×1 signal vector. $A\left(f_{j},\theta \right)=\left[a\left(f_{j},\theta_{1}\right),a\left(f_{j},\theta_{2}\right),\cdots ,a\left(f_{j},\theta_{K}\right)\right]$ is the array manifold matrix at the frequency point $f_{j}$, and $a\left(f_{j},\theta_{k}\right)\in C^{M\times 1}$ is the steering vector corresponding to the k-th signal, $a\left(f_{j},\theta_{k}\right)={\left[1,e^{-j2\pi f_{j}d\sin\theta_{k}/c},\cdots ,e^{-j2\pi f_{j}\left(M-1\right)d\sin\theta_{k}/c}\right]}^{T}$.

Equation (2) represents the mathematical model of the wideband array signal for a single snapshot. In practice, a whole period of time can be divided into _*L*_ equally spaced segments, provided that the duration of each segment exceeds the correlation time between signal and noise. This ensures the acquisition of multiple snapshots in the frequency domain. The mathematical model for the l-th snapshot can be expressed as

(3) $$x_{l}\left(f_{j}\right)=A\left(f_{j},\theta \right)s_{l}\left(f_{j}\right)+n_{l}\left(f_{j}\right),\;l=1,2,\cdots ,L$$

Given that the signal is uncorrelated with the noise, and different channel noises are uncorrelated, the covariance matrix for the j-th frequency point is

(4) $$R_{j}=E\left[x_{l}\left(f_{j}\right)x_{l}^{H}\left(f_{j}\right)\right]=A\left(f_{j},\theta \right)R_{s}A^{H}\left(f_{j},\theta \right)+\sigma_{n}^{2}I$$

where $E\left[\cdot \right]$ represents the expectation operation, $R_{s}=E\left[s_{l}\left(f_{j}\right)s_{l}^{H}\left(f_{j}\right)\right]$ is the signal covariance matrix, $\sigma_{n}^{2}$ denotes the noise power, and _I_ is the identity matrix. The superscript ${\left(\cdot \right)}^{H}$ indicates the conjugate transpose operation.

From Equation (4), we observe that $A\left(f_{j},\theta \right)$ depends on both the signal incidence angle θ and the frequency $f_{j}$. Despite the fixed angular information of the sources, the covariance matrix at each frequency differs due to frequency-dependent characteristics. This leads to a distinct mapping between the covariance matrix $R_{j}$ and the incidence angle θ for each sub-frequency band. General processing techniques, such as ISM and CSM, are commonly paired with traditional algorithms such as MUSIC or SR to perform DOA estimation. However, these model-driven strategies face significant limitations when applied to complex environments.

Machine learning methods establish a mapping relationship between the input and output through automatic data learning, which can overcome the limitations of traditional array models, and they can accurately estimate the DOAs of signal sources without requiring a complex mathematical model. For wideband signals, it is necessary to establish the mapping relationship between the array covariance matrix at each frequency point and the incident angle, i.e., $F=\left\{R_{1},R_{2},\cdots ,R_{J}\right\}\to {\left\{\theta_{k}\right\}}_{k=1}^{K}$, in order to obtain the angular information of the source. Although a mapping model has been given in [38], the dimensionality of the parallel input data grows with the number of sub-bands, resulting in a significant increase in the overall network training time.

## 3. The Proposed Method

### 3.1. Data Preprocessing

CSM is an effective method for handling broadband signals, as it has the advantages of low computational complexity, superior estimation accuracy, etc. The core of the CSM algorithm is the construction of a focusing matrix. In this paper, the TCT algorithm is adopted to design the focusing matrix to satisfy the minimization problem with the following constraints:

(5) $$\left\{\begin{array}{l} \underset{T(f_{j})}{\min}{\left\|P\left(f_{0}\right)-T\left(f_{j}\right)P\left(f_{j}\right)T^{H}\left(f_{j}\right)\right\|}_{F},\;j=1,2,\cdots ,J\\ T\left(f_{j}\right)T^{H}\left(f_{j}\right)=I \end{array}\right.$$

where $P\left(f_{0}\right)=A\left(f_{0},\theta \right)E\left[s_{l}\left(f_{0}\right)s_{l}^{H}\left(f_{0}\right)\right]A^{H}\left(f_{0},\theta \right)$ represents the denoised covariance matrix of the array output data at the reference frequency point, $f_{0}$ is the reference frequency point, $P\left(f_{j}\right)=A\left(f_{j},\theta \right)E\left[s_{l}\left(f_{j}\right)s_{l}^{H}\left(f_{j}\right)\right]A^{H}\left(f_{j},\theta \right)$ is the denoised covariance matrix of the array output data at the frequency point $f_{j}$, and ${\left\|\cdot \right\|}_{F}$ denotes the Frobenius norm of the matrix. In practice, $P\left(f_{j}\right)$ is estimated using $P\left(f_{j}\right)={\hat{R}}_{j}-{\hat{\sigma }}_{j}^{2}I$, where ${\hat{R}}_{j}=\frac{1}{L}\sum_{l=1}^{L}x_{l}\left(f_{j}\right)x_{l}^{H}\left(f_{j}\right)$ is the estimated value of $R_{j}$, and ${\hat{\sigma }}_{j}^{2}$ is the estimated noise power, i.e., the average of the small eigenvalues of the covariance matrix ${\hat{R}}_{j}$ at the frequency point $f_{j}$.

Reference [44] gives a solution to Equation (5), i.e.,

(6) $$T\left(f_{j}\right)=Q\left(f_{0}\right)Q^{H}\left(f_{j}\right)$$

where $Q\left(f_{0}\right)\in C^{M\times M}$ and $Q\left(f_{j}\right)\in C^{M\times M}$ are the eigenvectors of $P\left(f_{0}\right)$ and $P\left(f_{j}\right)$, respectively, and the columns are mutually orthogonal.

When multi-frequency data from the wideband signal are directly input into the SVR model, the training time increases significantly. In this paper, we employ a focusing technique to map the multi-frequency data to the reference frequency point. This approach reduces the input dimensionality of the model without compromising the effective information across all frequency bands. By applying Equation (6) to Equation (3), we obtain the focused data

(7) $$x_{l}^{0}\left(f_{j}\right)=T\left(f_{j}\right)\cdot x_{l}\left(f_{j}\right)=A\left(f_{0},\theta \right)\cdot s_{l}\left(f_{0}\right)+n_{l}^{0}\left(f_{j}\right)$$

where $x_{l}^{0}\left(f_{j}\right)$ and $n_{l}^{0}\left(f_{j}\right)=T\left(f_{j}\right)\cdot n_{l}\left(f_{j}\right)$ denote the receive data and the noise vector after focusing at frequency $f_{j}$ for the l-th snapshot, respectively. Since the focusing matrix $T\left(f_{j}\right)$ is a unitary matrix and satisfies the constraint of $T\left(f_{j}\right)T^{H}\left(f_{j}\right)=I$, the noise vector after focusing follows a Gaussian distribution with 0 mean and variance $\sigma_{n}^{2}$.

Using Equations (4) and (7), the focused covariance matrix can be calculated:

(8) $$\begin{array}{ll} R_{0} & =\frac{1}{J}\sum_{j=1}^{J}E\left[x_{l}^{0}\left(f_{j}\right)x_{l}^{0}{\left(f_{j}\right)}^{H}\right]\\ & =\frac{1}{J}\sum_{j=1}^{J}\left(E\left[T\left(f_{j}\right)\cdot x_{l}\left(f_{j}\right)x_{l}^{H}\left(f_{j}\right)T^{H}\left(f_{j}\right)\right]\right)\\ & =\frac{1}{J}\sum_{j=1}^{J}\left(T\left(f_{j}\right)R_{j}T^{H}\left(f_{j}\right)\right) \end{array}$$

In practice, $R_{0}$ can be estimated from _*L*_ snapshot data:

(9) $$\begin{array}{cc} {\hat{R}}_{0} & =\frac{1}{J}\sum_{j=1}^{J}\left(T\left(f_{j}\right){\hat{R}}_{j}T^{H}\left(f_{j}\right)\right)\\ & =\frac{1}{J}\sum_{j=1}^{J}\sum_{l=1}^{L}\left(T\left(f_{j}\right)x_{l}\left(f_{j}\right)x_{l}^{H}\left(f_{j}\right)T^{H}\left(f_{j}\right)\right) \end{array}$$

where $\hat{R}$ denotes the estimated values of **_R_**. The more accurate the estimation of **_R_**, the higher the training quality of the proposed model framework.

After the focusing operation, the data dimensionality of the wideband array signals can be reduced by a factor of J. Particularly for broadband or ultra-wideband signals, the proposed algorithm effectively reduces the storage requirements and computational complexity. By analyzing the structure of the covariance matrix ${\hat{R}}_{0}$, it is known that it is a conjugate symmetric matrix, and its diagonal elements are independent of the signal incidence angle. Therefore, the upper triangular elements of the output covariance matrix are extracted as input features for the model, which can be formulated as

(10) $$\eta ={\left[r_{12},\cdots ,r_{1M},r_{23},\cdots ,r_{2M},\cdots ,r_{(M-1)M}\right]}^{T}$$

where $r_{ij}={\hat{R}}_{0}\left(i,j\right)$ is the i-th row and j-th column element of ${\hat{R}}_{0}$.

In Equation (10), the complex-valued vector η is decomposed into its real and imaginary components and converted into a real-valued vector for subsequent processing. After normalization, the input feature vector $z_{0}$ is derived as

(11) $$z_{0}=\frac{{\left[ℜ\left(\eta^{T}\right),ℑ\left(\eta^{T}\right)\right]}^{T}}{{\left\|\eta \right\|}_{2}}$$

where ${\left\|\cdot \right\|}_{2}$ denotes the $l_{2}$ norm of the vector, $ℜ\left(\cdot \right)$ is the operation of taking the real part, and $ℑ\left(\cdot \right)$ is the operation of taking the imaginary part. The dimension of the input feature vector $z_{0}$ is $M\left(M-1\right)$, which remains constant at all times.

### 3.2. Optimization of SVR Parameters

SVR is a regression algorithm that maps data into a high-dimensional space and fits data by identifying an optimal hyperplane, thereby enabling accurate estimation of target values. It is capable of effectively addressing nonlinear problems, exhibiting strong generalization capability and demonstrating robustness to noise and outliers. In the SVR model, the regularization parameter C and the kernel function parameter γ are two critical hyperparameters that jointly determine the complexity and generalization performance of the model. The regularization parameter C controls the model complexity, striking a balance between training error and generalization capability. A larger C causes the SVR to overly focus on the accuracy of the training set, leading to overfitting. A smaller C may prevent the model from capturing the characteristics of the training data well, resulting in underfitting. γ is essential for mapping the input data into high-dimensional space. A smaller γ means a larger influence range for the data points, which can lead to underfitting. A larger γ means a smaller influence range for each data point, which may lead to overfitting. In Reference [45], PSO is employed to optimize the parameters of the SVR model, but the resulting computational burden remains significantly high, limiting its practical applicability. Therefore, this research introduces a genetic algorithm, which gradually approximates the optimal C and γ estimates by imitating the process of biological evolution and conducting extensive searches in the solution space. The detailed steps are described as follows.

#### 3.2.1. Initializing the Population

The purpose of parameter optimization is to enhance the estimation performance by adjusting the parameter combinations of the SVR model. The first step in implementing the genetic algorithm is population initialization, which involves randomly sampling the parameter space to generate initial candidate solutions within the predefined range of values for parameters C and γ. Each candidate solution is represented as a two-dimensional vector $\left[C_{i},\gamma_{i}\right],(i\leq m)$, where _*m*_ denotes the population size. The fitness of each parameter combination is evaluated via simulation experiments, and individuals meeting the criteria are added to the initial population set S. The iterative process proceeds until the population size attains the predefined threshold, completing the construction of the initialization stage.

#### 3.2.2. Calculating Adaptation

In the parameter optimization process, the fitness function serves to evaluate each parameter combination, thereby guiding the evolutionary direction of the GA. As shown in Equation (12), the fitness function designed in this study incorporates both the model prediction error and multi-objective detection metrics. This dual consideration enables effective evaluation of the relative quality of population individuals and drives the algorithm toward superior solutions.

(12) $$f=\sqrt{\frac{1}{NK}\sum_{n=1}^{N}\sum_{k=1}^{K}{\left[\theta_{k}\left(n\right)-{\hat{\theta }}_{k}\left(n\right)\right]}^{2}}$$

where N is the number of test samples, ${\hat{\theta }}_{k}\left(n\right)$ is the angle estimation value of the sample, and $\theta_{k}\left(n\right)$ is true angle of the sample. The smaller the value of f, the better the performance of the model after parameter optimization.

#### 3.2.3. Selection

**Figure 1:**
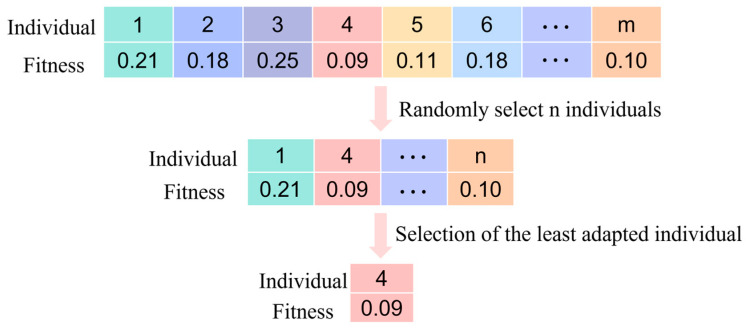
Selection operation.

As shown in Figure 1, the selection process of the genetic algorithm in this study involves a tournament method. In order to create a new generation of populations, the tournament selection strategy first compares n(n<m) individuals from population S and chooses the individuals with the lowest fitness to form part of the offspring population. This process is repeated until the new population size equals the original population size.

#### 3.2.4. Crossover

**Figure 2:**
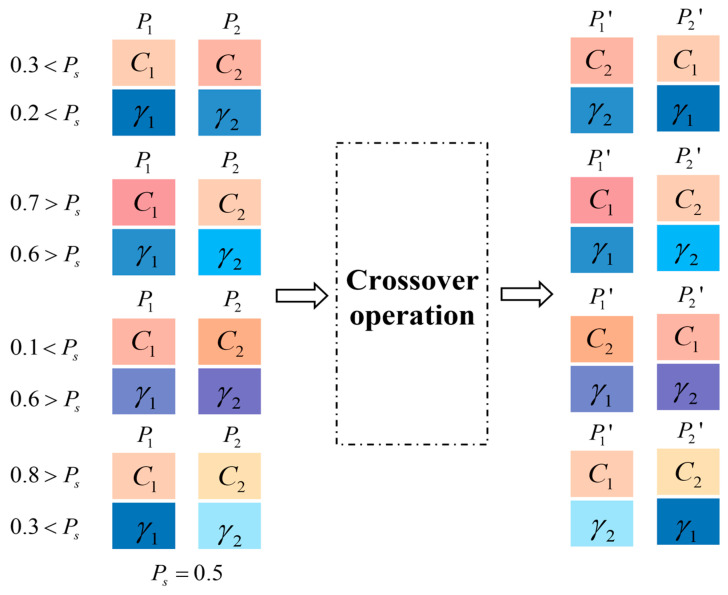
Crossover operation.

In this paper, we use the uniform crossover approach, which is shown in Figure 2. The crossover operation decides whether the genes at index i of the two chromosomes should be exchanged, depending on the exchange probability $P_{s}$, to generate a new offspring. Because the chromosome length for two parent individuals, $P_{1}=\{C_{1},\gamma_{1}\}$ and $P_{2}=\{C_{2},\gamma_{2}\}$, is _2_, there are only four crossover directions available. Through this method, the newly generated individuals inherit advantageous characteristics from their predecessors before the crossover, simultaneously introducing novel gene combinations. This mechanism not only preserves beneficial traits but also promotes genetic diversity within the population. As a result, it effectively mitigates the risk of premature convergence and reduces the chance of the algorithm getting trapped in local optima, thereby enhancing the global search capability of the optimization process.

#### 3.2.5. Mutation

**Figure 3:**
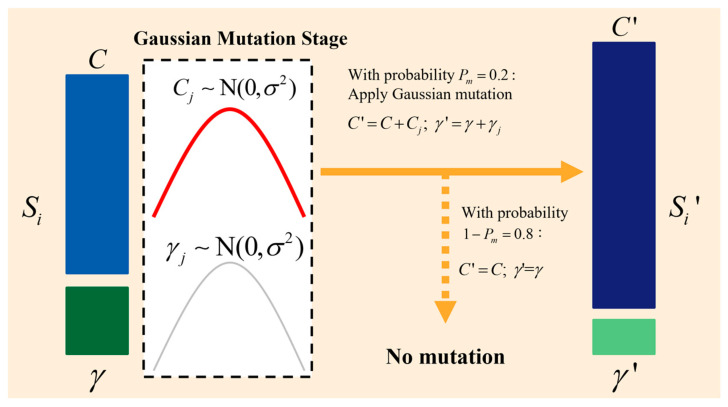
Variant operation.

In genetic algorithms, Gaussian mutation is a frequently used mutation operation that creates new individuals by randomly perturbing the chromosomes of the individuals in a way that follows a Gaussian distribution. This mutation operation can preserve population diversity to a certain degree and assist the algorithm in breaking out of the local optimum. As shown in Figure 3, for each individual $S_{i}$, a random number $r_{j}∼N(\mu ,\sigma^{2})$ is created, where $N(\mu ,\sigma^{2})$ is a Gaussian distribution with variance $\sigma^{2}$ and mean μ. *_σ_* is the standard deviation of the Gaussian mutation, which controls the strength of the mutation. A larger *_σ_* leads to a larger magnitude of the mutation, which increases the diversity of the population. A smaller *_σ_* leads to a more localized mutation, which helps to perform a fine-grained search in the neighborhood of the current solution search. Next, we choose whether the random number $r_{j}$ is added to the gene according to the mutation probability $P_{m}$ to obtain the mutated individual $S_{i}\prime$. After performing Gaussian mutation, the mutated parameter values are checked to determine whether they exceed the predefined bounds. If any parameter falls outside the allowable range, it is adjusted to the nearest boundary value. This ensures that all parameter values remain within the preset limits, maintaining the validity and feasibility of the solution space during the evolutionary process.

#### 3.2.6. Circulation

After mutation, the fitness of each individual in the population is calculated according to Equation (12), and the individual with the smallest fitness value is recorded. This evaluation–selection process is iteratively repeated, ensuring the continuous improvement of the population over successive generations. The iteration continues until a predefined termination condition is satisfied. Upon completion of the optimization process, the individual corresponding to the minimum fitness value is selected as the optimal estimation result of the C and γ parameters, thereby ensuring the best possible performance of the model.

### 3.3. Multiband Joint Direction-Finding Method Based on SVR

**Figure 4:**
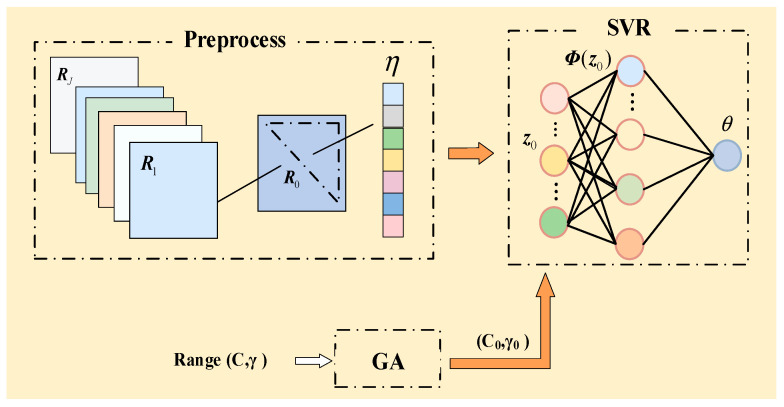
SVR model for multiband joint direction finding.

From Equation (4), it can be seen that the incident signal angle space $\Theta \subset R^{K}$ and the array covariance matrix space $R\subset C^{M\times M}$ are connected via a mapping function. After the data preprocessing in Section 3.1, the angle space is mapped to the feature vector $z_{0}$. There exists the mapping relation $G:\theta \to z_{0}$, and the DOA estimation can be regarded as the mapping of the angle space of sources from the input feature space of the array, $F:z_{0}\to \theta$, i.e., it is the inverse mapping of the mapping relation G. Building on this, the SVR parameters are optimized using the GA, and an SVR model for multiband joint direction finding is subsequently established, as illustrated in Figure 4. It is important to point out that the detail of the signal features varies with different numbers of array elements and bandwidths, which consequently leads to different optimization results of C and γ. This model achieves the inverse mapping from the input feature vector to the angle space. Firstly, the SVR model is trained with sample data to obtain $\hat{F}$. Subsequently, during the testing phase, the trained model $\hat{F}$ is employed to estimate the angles of the input wideband signal test samples with unknown directions.

The objective of the SVR model in the proposed algorithm is to learn a functional relationship between the input features used for training $z_{0}$ and the output $\hat{\theta }$:

(13) $$\hat{\theta }=\hat{F}\left(z_{0}\right)=w^{T}\Phi \left(z_{0}\right)+b$$

where $\hat{\theta }$ denotes the estimation of θ, $\Phi \left(\cdot \right)$ is a nonlinear mapping function that maps the input feature $z_{0}$ to a higher-dimensional space, and $\left\{w,b\right\}$ are the model parameters to be trained. Through the optimization of the objective function, the problem presented in Equation (13) can be equivalently transformed into

(14) $$\left\{\hat{w},\hat{b}\right\}=\underset{\left\{w,b\right\}}{argmin}\frac{1}{2}{\left\|w\right\|}^{2}+C\sum_{p=1}^{P}L_{\varepsilon }\left(\theta^{p},\hat{F}\left(z_{0}^{p}\right)\right)$$

where C is a regularization parameter, $L_{\varepsilon }\left(\cdot \right)\;$ denotes the loss function, $L_{\varepsilon }\left(\theta^{p},\hat{F}\left(z_{0}^{p}\right)\right)=\left\{\begin{array}{l} \left|\theta^{p}-\hat{F}\left(z_{0}^{p}\right)\right|-\varepsilon ,\left|\theta^{p}-\hat{F}\left(z_{0}^{p}\right)\right|>\varepsilon \\ 0,otherwise \end{array}\right.$, and $\left\{\left(z_{0}^{1},\theta^{1}\right),\left(z_{0}^{2},\theta^{2}\right),\cdots ,\left(z_{0}^{P},\theta^{P}\right)\right\}$ denotes _*P*_ sets of wideband signal training data.

To accommodate allowable prediction errors in the SVR model, the slack variables $\xi_{p}$ and ${\xi_{p}}^{\prime }$ are introduced, leading to the reformulation of Equation (14) as follows:

(15) $$\begin{array}{l} \underset{\left\{w,b,\xi_{p},\xi_{p}^{\prime }\right\}}{\min}\frac{1}{2}{\left\|w\right\|}^{2}+C\sum_{p=1}^{P}(\xi_{p}+\xi_{p}^{\prime })\\ s.t.\;\;\;\;\left(w^{T}\Phi \left(z_{0}^{p}\right)+b\right)-\theta^{p}\leq \varepsilon +\xi_{p}\\ \;\;\;\;\;\;\;\;\theta^{p}-\left(w^{T}\Phi \left(z_{0}^{p}\right)+b\right)\leq \varepsilon +\xi_{p}^{\prime }\\ \;\;\;\;\;\;\;\;\;\xi_{p}\geq 0,\xi_{p}^{\prime }\geq 0,p=1,2,\cdots ,P \end{array}$$

Similar to the derivation process in [38], the dual problem of Equation (15) can be obtained as follows:

(16) $$\begin{array}{l} L=\underset{\alpha_{p},\alpha_{p}^{\prime }}{\min}-\frac{1}{2}\sum_{p=1}^{P}\sum_{i=1}^{P}\left(\alpha_{p}^{\prime }-\alpha_{p}\right)\left(\alpha_{i}^{\prime }-\alpha_{i}\right)\kappa \left(z_{0}^{p},z_{0}^{i}\right)+\\ \;\;\;\;\;\;\;\;\;\;\;\;\;\;\;\;\;\sum_{p=1}^{P}\theta^{p}\left(\alpha_{p}^{\prime }-\alpha_{p}\right)-\sum_{p=1}^{P}\varepsilon \left(\alpha_{p}+\alpha_{p}^{\prime }\right)\\ \;\;\;\;\;\;\;\;s.t.\;\;\sum_{p=1}^{P}\left(\alpha_{p}-\alpha_{p}^{\prime }\right)=0\\ \;\;\;\;\;\;\;\;\;\;\;\;\;\;\;\;\;0\leq \alpha_{p},\alpha_{p}^{\prime }\leq \lambda ,p=1,2,\cdots ,P \end{array}$$

where $\alpha_{p},\alpha_{p}^{\prime }$ are Lagrange multipliers, and $\kappa (z_{0}^{p},z_{0}^{i})=e^{-\gamma {\left\|z_{0}^{p}-z_{0}^{i}\right\|}^{2}}={\left[\Phi (z_{0}^{p})\right]}^{T}\Phi (z_{0}^{i})$ is the kernel function. Obviously, Equation (16) is a quadratic programming problem, which can be solved by the sequential minimum optimization algorithm for the parameter ${\left\{\alpha_{p},\alpha_{p}^{\prime }\right\}}_{p=1}^{P}$. The final DOA estimation result of the proposed algorithm in this paper can be obtained as

(17) $$\hat{\theta }=w^{T}\Phi \left(z_{0}\right)+b=\sum_{p=1}^{P}\left(\alpha_{p}^{\prime }-\alpha_{p}\right)\kappa \left(z_{0}^{p},z_{0}\right)+b$$

As can be seen from Equation (17), the algorithm proposed in this paper takes the single-frequency-point data as the input feature of the network and the signal incidence angle as the output. This design enables the joint estimation of wideband signals across multiple frequency bands without significantly increasing the model complexity. By avoiding the direct input of multiband data, the algorithm effectively reduces the dimensionality and scale of the network model, thereby decreasing the training time and computational cost. Moreover, this approach ensures that the essential features of the wideband signal are preserved, thereby achieving high estimation accuracy alongside improved computational efficiency. An algorithm block diagram of the proposed method is given by Algorithm 1. $N_{epochs}$ represents the number of epochs used for the GA. $Model_{SVR}(C*,\gamma *)$ represents the trained SVR model. C* and γ* are the optimal parameter estimation results. upper(⋅) denotes taking the upper triangular part, and vec(⋅) is the vectorization operator.
**Algorithm 1:** Broadband signal processing and parameter optimization **Inputs**$:\;x_{m}\left(t\right)$J$,\;L,\;\{\theta_{i}\}$$,\;N_{epochs}$ **Outputs**$:\;Model_{SVR}(C*,\gamma *)$Sub-band decomposition using Equations (2) and (3): $x_{m}\left(t\right)\to \left\{x_{l}\left(f_{1}\right),x_{l}\left(f_{2}\right),\cdots ,x_{l}\left(f_{J}\right)\right\}$;Estimate the covariance matrix at frequency $f_{j}:\;{\hat{R}}_{j}=\frac{1}{L}\sum_{l=1}^{L}x_{l}\left(f_{j}\right)x_{l}^{H}\left(f_{j}\right)$;Construct the focusing matrix using Equation (6);TCT focusing: ${\hat{R}}_{0}=\frac{1}{J}\sum_{j=1}^{J}\sum_{l=1}^{L}\left(T\left(f_{j}\right)x_{l}\left(f_{j}\right)x_{l}^{H}\left(f_{j}\right)T^{H}\left(f_{j}\right)\right)$;Extract the feature vector: $z_{0}=\frac{{\left[ℜ\left(\eta^{T}\right),ℑ\left(\eta^{T}\right)\right]}^{T}}{{\left\|\eta \right\|}_{2}}$$,\;\eta =vec(upper({\hat{R}}_{0}))$;Genetic algorithm optimizes SVR parameters:      for $n_{epochs}=1$$\;\mathrm{to}\;N_{epochs}$            $\mathrm{Initialization}\;\left(C,\gamma \right)$;            $\overset{\wedge }{\theta }=Model_{SVR}(z_{0};C,\gamma )$;            Calculating adaptation using Equation (12);            Selection; Crossover; Mutation;            $\left(C_{n_{epochs}},\gamma_{n_{epochs}}\right)=\arg\underset{\left(C,\gamma \right)}{\min}\;f=\sqrt{\frac{1}{NK}\sum_{n=1}^{N}\sum_{k=1}^{K}{\left[\theta_{k}\left(n\right)-{\hat{\theta }}_{k}\left(n\right)\right]}^{2}}$;            $n_{epochs}=n_{epochs}+1$;      end      $\left(C*,\gamma *\right)=\arg\underset{\left(C_{n_{epochs}},\gamma_{n_{epochs}}\right)}{\min}\;f$;$\overset{\wedge }{\theta }=Model_{SVR}(z_{0};C*,\gamma *)$;**Return**  $Model_{SVR}(C*,\gamma *)$.

## 4. Performance Analysis

To demonstrate the effectiveness of the proposed algorithm and analyze its performance, a series of simulation experiments are conducted. Assuming that the receiving array is a ULA with six elements, the center frequency of the far-field wideband signal is 8 MHz, the bandwidth is 4 MHz, and the spacing of the elements is 15 m.

### 4.1. DOA Estimation Results

**Figure 5:**
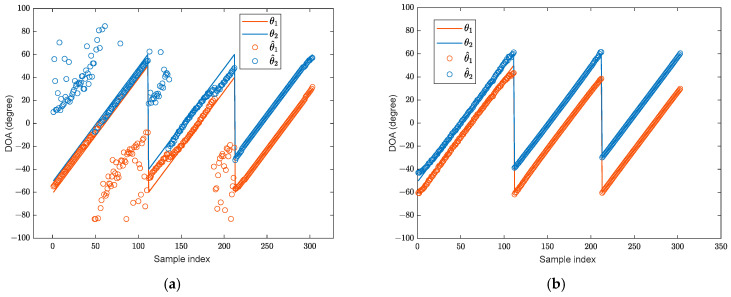

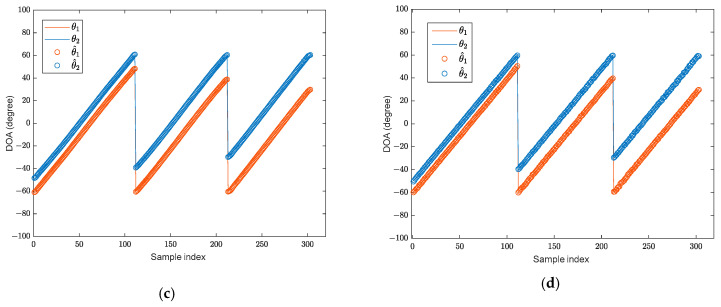
The direction-finding results: (**a**) RSS-MUSIC; (**b**) L1-SVD; (**c**) CWSVR; (**d**) proposed method.

**Figure 6:**
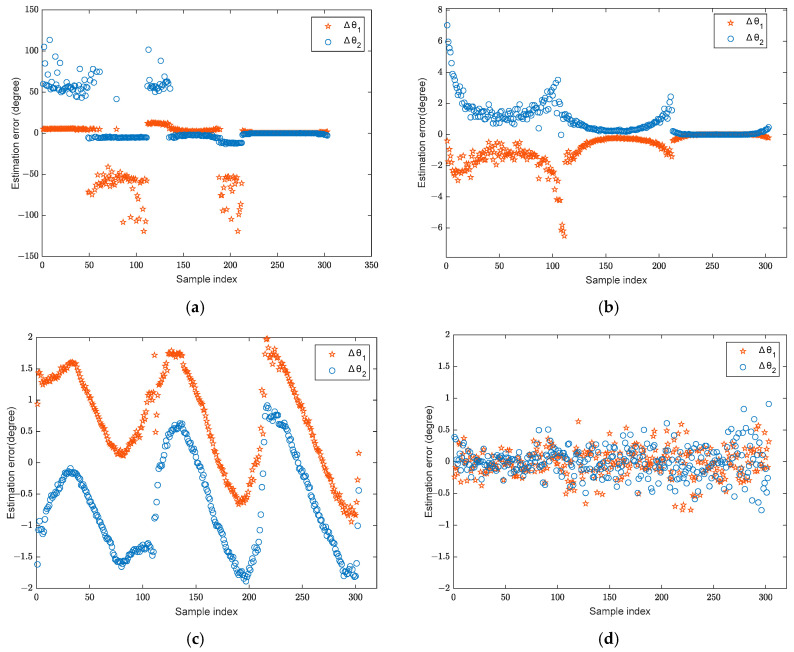
The DOA estimation error: (**a**) RSS-MUSIC; (**b**) L1-SVD; (**c**) CWSVR; (**d**) proposed method.

During the training stage, two broadband BPSK signals in the far-field are assumed, with their angular interval set to [9°, 11°,…, 35°], resulting in 14 distinct angle intervals in total. The signal sources are located within the angular range of [−60°, 60°] with an SNR of 10 dB, and the angular step is 1°. The snapshot number is set to 1024. In total, 1386 training samples are generated for model training. In the testing phase, the angle range of the two signals remains [−60°, 60°], while only three angle intervals [10°, 20°, 30°] are considered. The angular step is also 1°. The test signal adopts the same BPSK modulation and number of snapshots as the training stage. The SNR is 6 dB. A total of 303 test samples are generated. A comparative analysis is conducted between the DOA estimation results and errors of the proposed algorithm and those obtained using the RSS−MUSIC, L1−SVD, and CWSVR algorithms. Here, the estimation error is used as the evaluation metric, and it is defined as the difference between the estimated angle and the true value. The DOA estimation results are shown in Figure 5 (the solid lines represent the true DOAs), and the corresponding errors are shown in Figure 6. The predicted angle of the RSS-MUSIC method is determined using conventional beamforming, and the other experiments are equivalent.

The proposed method has high accuracy, as shown in Figure 6, and the majority of its estimation errors are concentrated within [−0.5°, 0.5°]. The CWSVR method also achieves favorable estimation performance across all signal intervals. However, its estimation errors are consistently larger than those of the proposed algorithm, remaining within _2°_. The L1-SVD method exhibits larger errors when the signal angular intervals are small, while its estimation performance improves as the intervals increase. The performance of the RSS-MUSIC method is influenced by the predicted angle, resulting in a wider fluctuation in estimation errors with small angular intervals.

### 4.2. Resolution Performance

**Figure 7:**
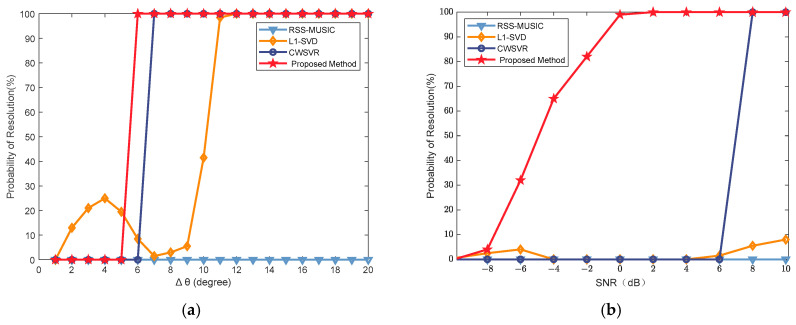
The resolution performance of two signals: (**a**) varying angular spacing; (**b**) varying SNR.

This experiment mainly analyzes the resolution performance of the proposed algorithm. Here, the definition of a successful resolution is as follows: a simulation trial is considered successful if the absolute error between all estimated signal angles and the true values remains within _1°_. Otherwise, it is regarded as a failure. In the case of two far-field wideband signals at *SNR* = 8 dB, the incident angle of the first signal is fixed at $\theta_{1}=0{}^{\circ}$, while the angle of the second signal is set to $\theta_{2}=\theta_{1}+\Delta \theta$. Δθ is the interval between the two angles, where the angular separation is $\Delta \theta \in \left(1{}^{\circ},2{}^{\circ},\cdots ,20{}^{\circ}\right)$. Each angular interval undergoes 200 Monte Carlo trials to derive the resolution probability versus the angular separation curve, as presented in Figure 7a. Additionally, with fixed signal positions $\theta_{1}=0{}^{\circ}$ and $\theta_{2}=7{}^{\circ}$, the SNR is systematically varied from −10 dB to 10 dB in 2 dB increments, producing the corresponding resolution probability versus SNR curve shown in Figure 7b.

As can be seen in Figure 7, with the increase in the angular interval and SNR, the resolution probability of all methods is in an increasing trend, and the successful resolution probability of the proposed algorithm is the first to reach 100%, which is higher than that of the other methods. The CWSVR method ranks second in performance. In the case of low SNR, the resolution performance of other methods is relatively poor, and the proposed method in this paper can still maintain a certain resolution probability. The L1-SVD method reaches 100% only when the angular separation exceeds _11°_. It can be seen that the probability of successful resolution is very low when the angular interval is _7°_, and there is no great improvement in the resolution probability even if the SNR is enlarged. The performance of the RSS-MUSIC method is highly sensitive to the predicted angle. When the predicted angle is inaccurate, the performance significantly degrades. It can be seen in Figure 7 that the RSS−MUSIC method is almost unable to successfully discriminate the signal under the same conditions.

### 4.3. Statistical Performance

**Figure 8:**
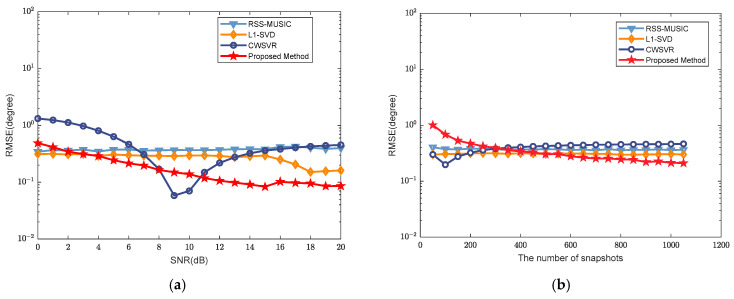
The estimation performance of two signals: (**a**) varying SNR; (**b**) varying number of frequency-domain snapshots.

This experiment analyzes the statistical performance of the proposed algorithm, which is evaluated using the root mean square error (RMSE), which is defined as

(18) $$\mathrm{RMSE}=\sqrt{\frac{1}{moto*K}\sum_{t=1}^{moto}\sum_{k=1}^{K}{\left({\hat{\theta }}_{k,t}-\theta_{k}\right)}^{2}}$$

where moto denotes the number of Monte Carlo iterations, ${\hat{\theta }}_{k,t}$ is the estimated angle of the k-th target in the t-th Monte Carlo experiment, and $\theta_{k}$ is the true angle of the *k*-th target. It is assumed that there are two wideband signals in the far-field space coming from angles of −9.56°+ζ and 15.72°+ζ, respectively. ζ takes a random value in the range of $\left[-0.5,\;0.5\right]$. The number of frequency-domain snapshots is fixed at 1024. The SNR increases from 0 dB to 15 dB in steps of 1 dB, and 500 Monte Carlo experiments are carried out for each SNR value. The resulting RMSE curve of the angle estimation versus the SNR is shown in Figure 8a. In addition, the SNR is fixed at 6 dB. The number of frequency-domain snapshots increases from 10 to 1050 in steps of 50, and 500 Monte Carlo experiments are performed for each number of frequency-domain snapshots. The resulting RMSE curve of the angle estimation versus the number of frequency-domain snapshots is shown in Figure 8b.

In Figure 8a, it can be seen that the RSS-MUSIC method can obtain better estimation performance when the angular interval is larger. RSSMUSIC has a smaller RMSE at a low SNR, but its statistical performance no longer improves as the SNR increases. The RMSE of the L1-SVD method is smaller than that of RSSMUSIC. The CWSVR method relies more on the training data and has a certain tendency of overfitting. When the SNR is greater than 3 dB, the RMSE of the proposed algorithm is lower than those of other methods. Furthermore, it achieves notable advantages in high-SNR scenarios with superior estimation accuracy. Additionally, minor fluctuations can be observed in the RMSE curve, particularly in the SNR range from 14 dB to 16 dB. This is attributed to the SNR mismatch between the training and testing data. From Figure 8b, it can be seen that the RMSE of the proposed algorithm decreases with the increase in the number of frequency-domain snapshots. When the number of frequency-domain snapshots exceeds 350, the proposed algorithm achieves the lowest RMSE compared to the other algorithms under the same conditions, demonstrating its superior performance.

### 4.4. Generalization Performance

**Figure 9:**
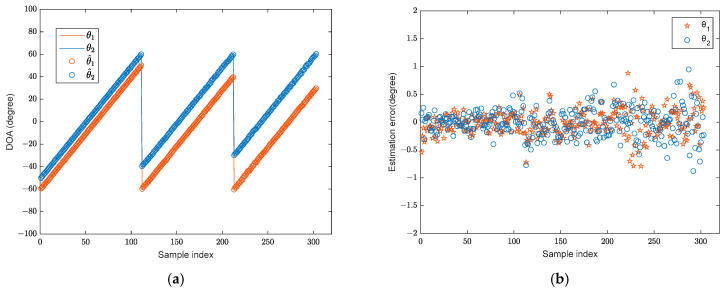
The direction-finding results and errors of the LFM signal with the proposed algorithm: (**a**) direction-finding results; (**b**) estimation error.

This experiment is designed to analyze the generalization performance of the proposed algorithm by assessing its DOA estimation accuracy under conditions where the signal types in the testing dataset differ from those in the training dataset. Maintaining the same training configuration as that in Section 4.1, the testing phase employs signal intervals of [10°, 20°, 30°] and two-signal angle ranges of [−60°, 60°] with _1°_ increments, generating a total of 303 test samples. The test signal is an LFM signal with the same bandwidth and an SNR of 6 dB. Figure 9 shows the DOA estimation results and corresponding errors obtained by the proposed algorithm.

Figure 9 shows that, when trained on BPSK signals, the proposed algorithm produces DOA estimation results for LFM signals that closely match the true signal angles across all signal intervals. The estimated angles almost coincide with the actual values, which demonstrates the robustness of the algorithm to variations in signal types. Moreover, the majority of estimation errors remain within $\left[-0.5{}^{\circ},\;0.5{}^{\circ}\right]$, indicating strong generalization ability and high estimation accuracy even when the signal type in the testing phase differs from that in the training phase. These results validate the adaptability and practical applicability of the proposed method in complex and diverse signal environments.

### 4.5. Comparison of Training and Testing Times

**Figure 10:**
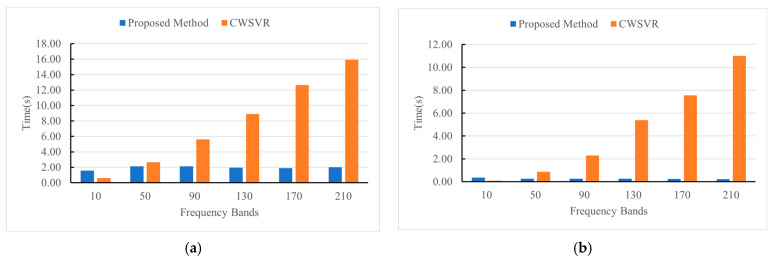
The variations in training time and testing time with the number of sub-bands: (**a**) training time; (**b**) testing time.

The primary objective of the proposed algorithm is to reduce the computational complexity of the wideband SVR model, thereby significantly decreasing its training time. To validate the efficiency improvement, we conduct a comparative analysis with the CWSVR method. An ASUS computer with an Intel(R) Core (TM) I7-9750 CPU is used as the simulation platform. Figure 10 illustrates the variation in training time and average testing time of 500 test samples as the number of sub-bands increases.

As shown in Figure 10, the training and testing times of the CWSVR method rise with the number of sub-bands. In contrast, the training and testing times of the proposed algorithm remain stable. When the number of sub-bands exceeds 40, our algorithm offers significant advantages in both training and testing times. This is attributed to the fact that the input feature dimension in the proposed algorithm does not increase with the number of sub-bands. As a result, the scale of the SVR model remains manageable, which not only reduces the computational complexity but also contributes to improved performance. This approach ensures both computational efficiency and effective handling of multiband signals, thereby facilitating faster training and enhancing the accuracy of the DOA estimation. It is worth noting that as the sample size increases, the efficiency advantage of the proposed method becomes more apparent.

## 5. Conclusions and Discussion

This paper establishes an effective SVR-based framework for DOA estimation, realizing joint multiband processing of wideband signals. On the one hand, this method reduces the input scale of the SVR model by converting the parallel input of multiple frequency points into single-frequency inputs. Consequently, the input dimension remains constant regardless of the number of sub-bands, thereby leading to a reduction in the training time for the model. On the other hand, a genetic algorithm is employed to optimize the SVR model parameters, enhancing its fitting and generalization capabilities and, thereby, improving the estimation performance for wideband signals. The simulation results demonstrate that the proposed approach achieves higher estimation accuracy and better angle resolution compared to the existing typical algorithms, alongside a significant reduction in training time. In the future, we plan to incorporate multi-task learning or multi-output regression into the proposed method, allowing the SVR model to simultaneously output the angle estimations of multiple sources and further enhance the estimation accuracy, especially under low-SNR and limited snapshot conditions.

## Data Availability

The original contributions presented in this study are included in the article. Further inquiries can be directed to the corresponding author.
